# Evaluation of a Well-Established Task-Shifting Initiative: The Lay Counselor Cadre in Botswana

**DOI:** 10.1371/journal.pone.0061601

**Published:** 2013-04-09

**Authors:** Jenny H. Ledikwe, Mable Kejelepula, Kabelo Maupo, Siwulani Sebetso, Mothwana Thekiso, Monica Smith, Bagele Mbayi, Nankie Houghton, Kabo Thankane, Gabrielle O’Malley, Bazghina-werq Semo

**Affiliations:** 1 Department of Global Health, University of Washington, Seattle, Washington, United States of America; 2 Botswana International Training and Education Center for Health (I-TECH), Gaborone, Botswana; 3 Botswana Ministry of Health, Gaborone, Botswana; 4 United States Centers for Disease Control and Prevention, Gaborone, Botswana; Indiana University, United States of America

## Abstract

**Background:**

Evidence supports the implementation of task shifting to address health worker shortages that are common in resource-limited settings. However, there is need to learn from established programs to identify ways to achieve the strongest, most sustainable impact. This study examined the Botswana lay counselor cadre, a task shifting initiative, to explore effectiveness and contribution to the health workforce.

**Methods:**

This evaluation used multiple methods, including a desk review, a national lay counselor survey (n = 385; response = 94%), in-depth interviews (n = 79), lay counselors focus group discussions (n = 7), lay counselors observations (n = 25), and client exit interviews (n = 47).

**Results:**

Interview and focus group data indicate that lay counselors contribute to essentially all HIV-related programs in Botswana and they conduct the majority of HIV tests and related counseling at public health facilities throughout the country. Interviews showed that the lay counselor cadre is making the workload of more skilled health workers more manageable and increasing HIV acceptance in communities. The average score on a work-related knowledge test was 74.5%. However for 3 questions, less than half answered correctly. During observations, lay counselors demonstrated average competence for most skills assessed and clients (97.9%) were satisfied with services received. From the survey, lay counselors generally reported being comfortable with their duties; however, some reported clinical duties that extended beyond their training and mandate. Multiple factors affecting the performance of the lay counselors were identified, including insufficient resources, such as private counseling space and HIV test kits; and technical, administrative, and supervisory support.

**Conclusion:**

Lay counselors are fulfilling an important role in Botswana's healthcare system, serving as the entry point into HIV care, support, and treatment services.

**Recommendation:**

For this and other similar task shifting initiatives, it is important that lay counselors' responsibilities are clear and that training and support are adequate to optimize their effectiveness.

## Introduction

Worldwide, there is a chronic shortage of trained health workers. In 2006, the World Health Organization (WHO) estimated that more than 4 million health workers were needed to meet the global shortfall.[1] This shortage is even more pronounced in resource-limited settings, particularly those hardest hit by the HIV epidemic. The need for skilled health professionals is a concern not only for ensuring access to HIV prevention, care, and treatment initiatives, but also for reaching health-related Millennium Development Goals. One strategy recommended by WHO for meeting human resources needs in the health sector is task shifting.[2]

Task shifting is the rational redistribution of tasks among workforce teams.[2] It is not a new concept and is common in many high-income countries in which lower-level cadres with appropriate training and supervision perform tasks delegated or shifted from higher-level cadres.[3]–[5] There is a substantial and growing body of literature supporting the use of task shifting in middle-income and low-income countries. While many of these initiatives have focused on HIV/AIDS programs,[6]–[23] task shifting is being implemented to address many other health conditions including tuberculosis,[24], [25] mental health disorders,[26]–[29] surgical services,[30]–[33] physical rehabilitation,[34] hypertension, and diabetes.[35], [36]

Multiple studies support the use of task shifting to strengthen HIV/AIDS programs. These include studies demonstrating acceptance and feasibility.[7], [11], [17], [19], [37] Studies have found that task shifting can increase access to HIV services [23] and increase efficiencies in service delivery.[21] Studies have also found that provider performance for duties delivered through various task shifting interventions yielded acceptable or improved quality of care and health outcomes.[12]–[14], [16], [22] In some instances, however, quality of care has been compromised in the absence of close monitoring of some task shifting initiatives.[8], [20] Additionally, modeling exercises suggest that task shifting can result in cost savings.[6] Even though much of these data are from short-term investigations or studies with a limited scope, there is little question about whether task shifting can play a vital role in strengthening the health workforce.[1], [2], [9], [38]

While evidence supports task shifting to address health worker shortages, there is a need to learn from large-scale, established programs to identify ways to achieve the largest, most sustainable impact. Several recent literature reviews have highlighted this gap in the literature and accentuated the need to better understand how to make the best use of the task-shifting approach [38] and how to use task shifting to achieve the strongest impact.[9] Another review emphasizes the need to address information gaps related to how task shifting can be implemented to ensure quality of care.[39]

The lay counselor cadre in Botswana can provide valuable information to address these gaps in the literature. In 1999, Botswana started Africa's first national program for prevention of mother-to-child transmission of HIV (PMTCT), which was followed by the implementation of the first national antiretroviral (ARV) program in Africa.[40], [41] In 2001, a new cadre of health worker, lay counselors, was created to assist nurses and midwives by providing counseling and support to women in antenatal and postnatal care. The lay counselors were also tasked with supporting the national ARV program. The cadre has been composed of individuals with a senior/high school certificate who underwent 4 to 6 weeks of training. They are based at public hospitals and clinics throughout the country.

This study examined the role of lay counselors in the provision of HIV services in Botswana's health facilities from 2002 to 2010 to identify factors related to the effectiveness of the cadre and their contribution to the health workforce. Specifically, the objectives of this evaluation were to: 1) describe the demographic characteristics and duties of the lay counselor cadre; 2) examine the performance of the lay counselors in terms of their knowledge, skills, and their contribution to the health workforce; and 3) explore factors related to the performance of the cadre.

## Methods

Multiple methods were used to conduct this evaluation. As summarized in Table 1, this included a desk review; a national survey of the lay counselors; in-depth interviews at the national, district, and facility levels; focus group discussions with lay counselors; observations of lay counselors carrying out their duties; client exit interviews; and secondary data analyses. This research was approved by the Botswana Health Research and Development Committee. The study was conducted by the International Training and Education Center for Health (I-TECH), which is a collaboration between the University of Washington and University of California, San Francisco under the guidance of a technical working group comprised of healthcare stakeholders. All participants provided signed informed consent.

**Table 1:** Summary of data collection methods used in the evaluation of the lay counselor cadre in Botswana.

Data collection method	Sample Description	Sample size (n)
Desk review	Documents relevant to the development and implementation of the cadre that were collected from members of the reference group, key informants, and lay counselors; which included training materials, job descriptions, presentations, and monitoring and evaluation tools	58 documents
National survey	All lay counselors at public health facilities in the country	385
Key informant interviews	National level: Purposeful sample of individuals involved in the development, administration, or training of lay counselors	17
	District level: Purposeful sample of district coordinators overseeing health programs supported by the lay counselors, including the district health team leadership as well as coordinators of the following programs: prevention of mother-to-child HIV transmission, HIV testing and counseling, Antiretroviral drug	23
	Facility level: Purposeful sample of healthcare workers closely involved with lay counselor, including individuals from each of the following cadres: physician, matron, nurse, midwife, and social worker	39
Focus group discussions	Purposeful sample of lay counselors from facilities in 7 districts	7 focus group discussions (76 lay counselors)
Counseling observations	Lay counselors at 3 facilities in each of 7 districts	25 lay counselors observed providing services to 47 clients
Client exit interviews	Clients participating in the counseling observation sessions	47

For the national survey, a self-administered questionnaire was distributed to all lay counselors at public health facilities in the country. Of 408 lay counselors, 385 returned their surveys (response rate = 94%). The questionnaire contained items related to demographics, duties, human resources, training, support, and job performance as well as a work-related knowledge test based on material covered during the pre-service training.

Seventy-nine in-depth interviews with health workers were conducted with a purposively-selected stratified sample at the national-level (n = 17), district-level (n = 23), and facility-level (n = 39). These were semi-structured interviews. At the national-level, interviews were held with individuals involved in the development, administration, or training of the lay counselor cadre. District-level interviews were conducted in seven districts purposely selected to obtain a mixture of two urban, three semi-urban, and two rural districts with a high number of lay counselors. This represented 29% of all health districts in the country (7/24). Interviews were held with district coordinators overseeing the health programs supported by the lay counselors, as well as with individuals who supervise and support the lay counselors, such as PMTCT focal persons, nurses, social workers, and doctors. Facility-level interviews were conducted at two facilities within each of the seven districts with health workers closely involved with lay counselors. All interviews were conducted face-to-face, with a rapporteur present to take notes. With permission, the interviews were captured with a digital voice recorder and transcribed.

Focus group discussions (FGDs) with lay counselors were held in each of the seven districts described above. This was used to compliment the quantitative information collected from the national survey. In total, 76 lay counselors participated in the FGDs, which represented19% of the cadre (76/408). During these sessions, in-depth information was collected about the lay counselor duties, pre-service and in-service training, support, and job performance. Each of the FGDs was captured with a digital voice recorder and transcribed.

Direct observations of lay counselors carrying out their duties were conducted by experienced nurse counselors in the same seven districts in which interviews and FGDs took place. Stratified random sampling was used to select three facilities in each of the seven districts to ensure that a hospital, clinic, and health post were included (if there was no health post in a district, a second clinic was selected). Lay counselors at each facility were asked to participate in the study. This encompassed one to three lay counselors per facility. Observations were conducted with 47 clients who consented to be observed. The observational assessment tools and procedures were adapted from those developed by UNAIDS,[42] which have been used in a similar setting.[43] The two nurse counselors who conducted the observations were trained on the tool to ensure consistent administration. They were not affiliated with any of the data collection facilities.

Client exit interviews were conducted to assess patient satisfaction with care by the same nurse counselor who observed the counseling sessions. Only clients whose counseling sessions were observed were included in the exit interviews (n = 47). A semi-structured questionnaire to elicit close-ended responses on clients' feelings about the services received that day was used. To help minimize social desirability influences, the nurse counselors who conducted the interviews emphasized that the purpose of this activity was for program improvement.

### Data analysis

Qualitative data included information from the desk review, key informant interviews, and focus group discussions as well as open-ended questions from the national survey and exit interviews. Transcripts were coded using ATLAS.ti v6.0 software for thematic analysis. Given that little information was available related to the day-to-day activities of the lay counselors or their performance; a grounded theory approach was taken as an inductive strategy for characterizing the performance of the cadre. Quantitative data included data from the national survey as well as data collected as part of the observations, exit interviews, and district-level routine HIV testing and counseling data. The survey data were entered into an Access database using a two-pass data verification process and analyzed using SPSS v15.0 software. Simple univariate analyses were used to describe the data. Paired t-tests were used to compare comfort-level ratings for job duties within and outside of the lay counselors' job description. Pearson correlations were used to compare years of experience as a lay counselor with comfort-rates relative to job duties.

## Results

### Profile of the lay counselor cadre

Data from the national survey indicated that the lay counselors ranged in age from 20 to 45 years, with a mean age±standard deviation (SD) of 30.6±4.0 years. Table 2 summarizes the demographic characteristics of the cadre. The majority were female (77.7%). Two-thirds of the respondents (66.2%) had worked as a lay counselor for five or more years.

**Table 2:** Demographic characteristics of the Botswana lay counselor cadre (n = 385).

Characteristic	N (%)
Age group (mean±SD = 30.6±4.0 years)	
20–24 years	17 (4.4%)
25–29 years	142 (36.9%)
30–34 years	152 (39.5%)
35–39 years	57 (14.8%)
40–44 years	8 (2.1%)
≥ 45 years	1 (0.3%)
No response	8 (2.1%)
Sex	
Male	86 (22.3%)
Female	299 (77.7%)
Marital status	
Single	298 (77.4%)
Married	63 (16.4%)
Divorced	1 (0.3%)
Living with partner	20 (5.2%)
No response	3 (0.8%)
Number of children	
0	99 (25.7%)
1	157 (40.8%)
2	92 (23.9%)
3	20 (5.2%)
4+	7 (1.8%)
No response	10 (2.6%)
Employment duration (Mean±SD = 5.1±2.4)	
≤1–2 years	85 (22.1%)
3–4 years	45 (11.7%)
5–6 years	99 (25.7%)
7–8 years	156 (40.5%)

### Training

On the national survey, most lay counselors (63.8%) reported that they had felt prepared to perform their duties following the 4–6 week pre-service training. The main weakness identified related to pre-service training during the FGDs and the interviews was that lay counselors and healthcare workers did not feel the training adequately addressed the lay counselors' added duties as the national HIV response expanded. There were, however, multiple in-service trainings conducted to supplement the pre-service training. During national-level interviews, it was reported that the in-service trainings were conducted, in part, to provide training related to new duties to be conducted by the lay counselors. Most (91.7%) of the lay counselors surveyed indicated that they had attended an in-service training, with 67.0% reporting attending at least one in-service training during the past year, and 43.4% reporting attendance at two or more trainings in the past year. When asked to rate how prepared they felt to perform their duties after attending in-service trainings, 88.5% indicated they felt prepared or very prepared. Two limitations of the in-service training were identified during FGDs and interviews: 1) not all lay counselors were able to participate in the trainings since resources were often not available for the entire cadre of lay counselors to participate, and 2) trainings were short in duration, lacked depth, and did not always match training needs.

### Knowledge and skill

**Figure 1:**
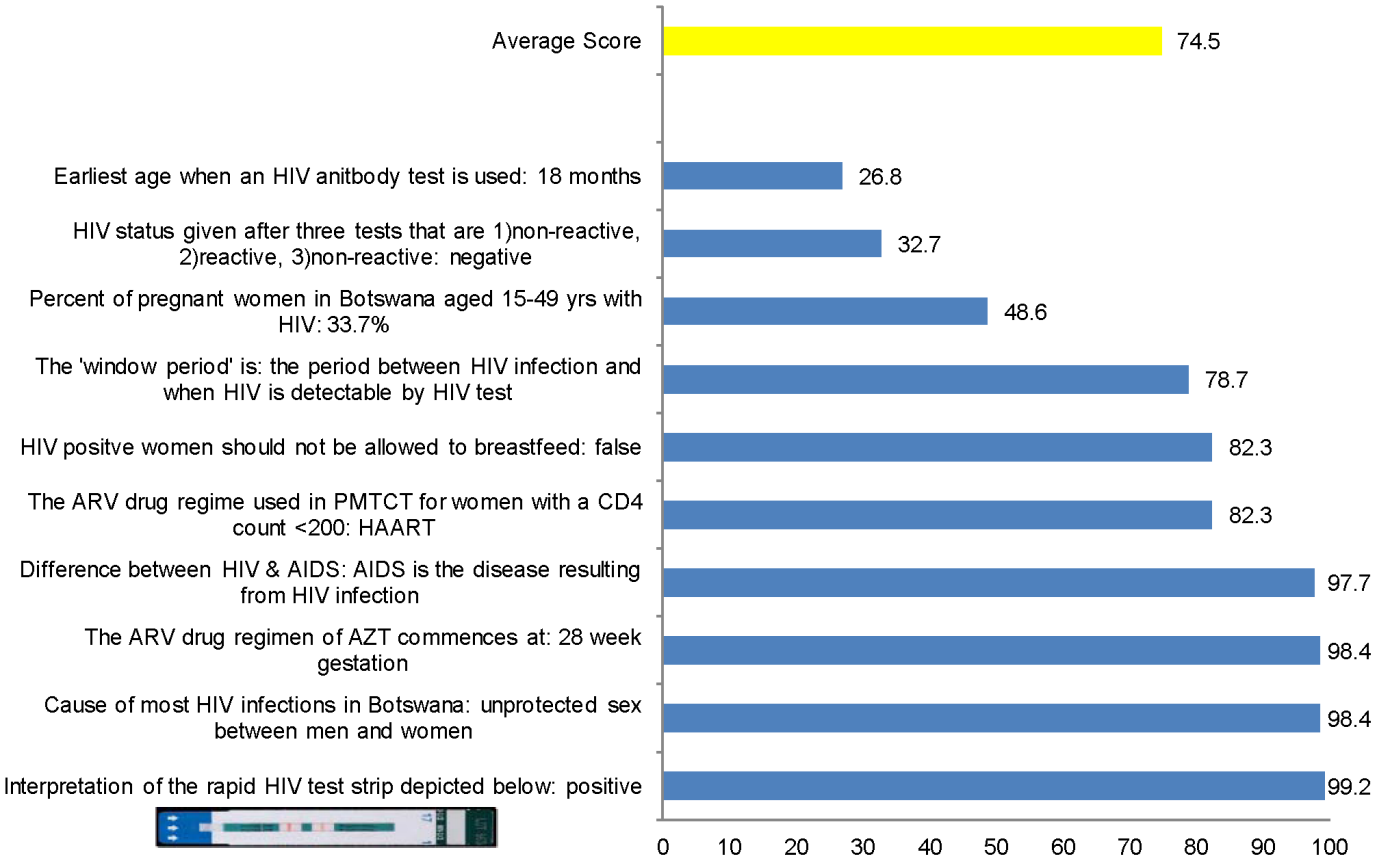
Percent score (correct responses) on 10-item knowledge test from the national survey (n = 385 lay counselors).

The average score on a 10-item knowledge test included in the national survey on topics related to their work was 74.5% (Figure 1). More than 50% of the lay counselors provided incorrect answers to the questions related to the earliest age at which an HIV antibody test can be used, to the interpretation of discordant HIV test results, and to the prevalence of HIV among women in the country. In-person observations were conducted with 25 lay counselors providing services to 47 clients. As presented in Table 3, counseling skills were rated using a 3-point scale (1 = unsatisfactory, 2 = average, 3 = high competence). Mean scores were 2.0 or higher for most competencies. During the observation session, 12 lay counselors were observed administering 20 HIV tests. Specimen collection and processing was done correctly for all 20 tests, however, the lay counselors did not wait for the designated amount of time before reading the test during two observations (10%). In one instance (5%), the test was not interpreted correctly. Test results were recorded improperly or not recorded for 6 of the 20 tests (30%). During national-level interviews, it was clarified that lay counselors performing HIV tests undergo bi-annual proficiency testing to maintain HIV testing certification, with supportive supervision provided by the national quality assurance lab as needed.

**Table 3:** Mean scores from observation sessions conducted with 25 lay counselors.^1^

Skill area	Mean score±SD
Basic counseling skills (n = 31 observations of 20 lay counselors)	
Interpersonal relationship skills and attitudes	2.4±0.4
Information gathering skills	1.9±0.4
Information giving skills	2.3±0.3
Skills in dealing with special circumstances	± 1.1
Pre-test counseling skills (n = 25 observations of 15 lay counselors)	
Introduction and correction of misperceptions related to HIV	2.0±0.5
Personal risk assessment developed with client	2.0±0.5
Shares information about HIV test	2.3±0.8
Assesses views and capacity to cope	2.1±0.8
Discussion of sharing results with someone	1.9±0.8
Discusses issues related to PMTCT	1.4±1.3
Post-test counseling: HIV negative results (n = 17 observations of 12 lay counselors)	
Review issues from pre-test counseling	2.9±0.3
Gives results clearly and simply	2.9±0.3
Gives time for consideration and discussion of results	2.1±0.8
Ensures client understands meaning of results	2.1±0.7
Addresses concerns that arise	2.3±0.9
Discusses the importance of staying negative	2.5±0.7
Develops a risk reduction plan	2.1±1.0
Counseling on infant feeding for HIV positive women (n = 17 observations of 11 lay counselors)	
Clear explanation of infant feeding recommendations	2.9±0.2
Discussion of risks benefits and difficulties of exclusive formula feeding and exclusive breast feeding	2.7±0.6
Discussion of risks of mixed feeding	2.4±0.8
AFASS assessment^2^	1.9±0.9
Assist with making feeding choice	2.5±0.6
Discuss obstacles that may be faced	2.2±0.8
Discuss support systems at home	2.2±1.0
Demonstration or practice with infant feeding option	2.3±0.8
Assurance the counselor is available for future support	2.1±0.1

1 Means calculated based on each observation using a 3-point scale in which 1 = unsatisfactory, 2 = average, and 3 = high competence

2 Affordable, Feasible, Accessible, Safe, and Sustainable assessment for appropriateness of replacement feeding of infants born to HIV positive mothers

During the exit interviews, most clients (n = 46; 97.9%) reported being satisfied with the counseling and testing services received and that they felt comfortable returning for counseling in the future (n = 46; 97.9%).

### Job duties

One of the main duties listed in the job description for the lay counselors is counseling. This includes counseling before and after administration of an HIV test, on-going supportive counseling for HIV positive clients, infant feeding counseling, and HIV prevention counseling. In addition to counseling, other duties on the job description include monitoring and evaluation activities such as compiling HIV test results and registering new antenatal clients, rapid HIV test administration, and client and community education such as providing health talks and home visits.

**Figure 2:**
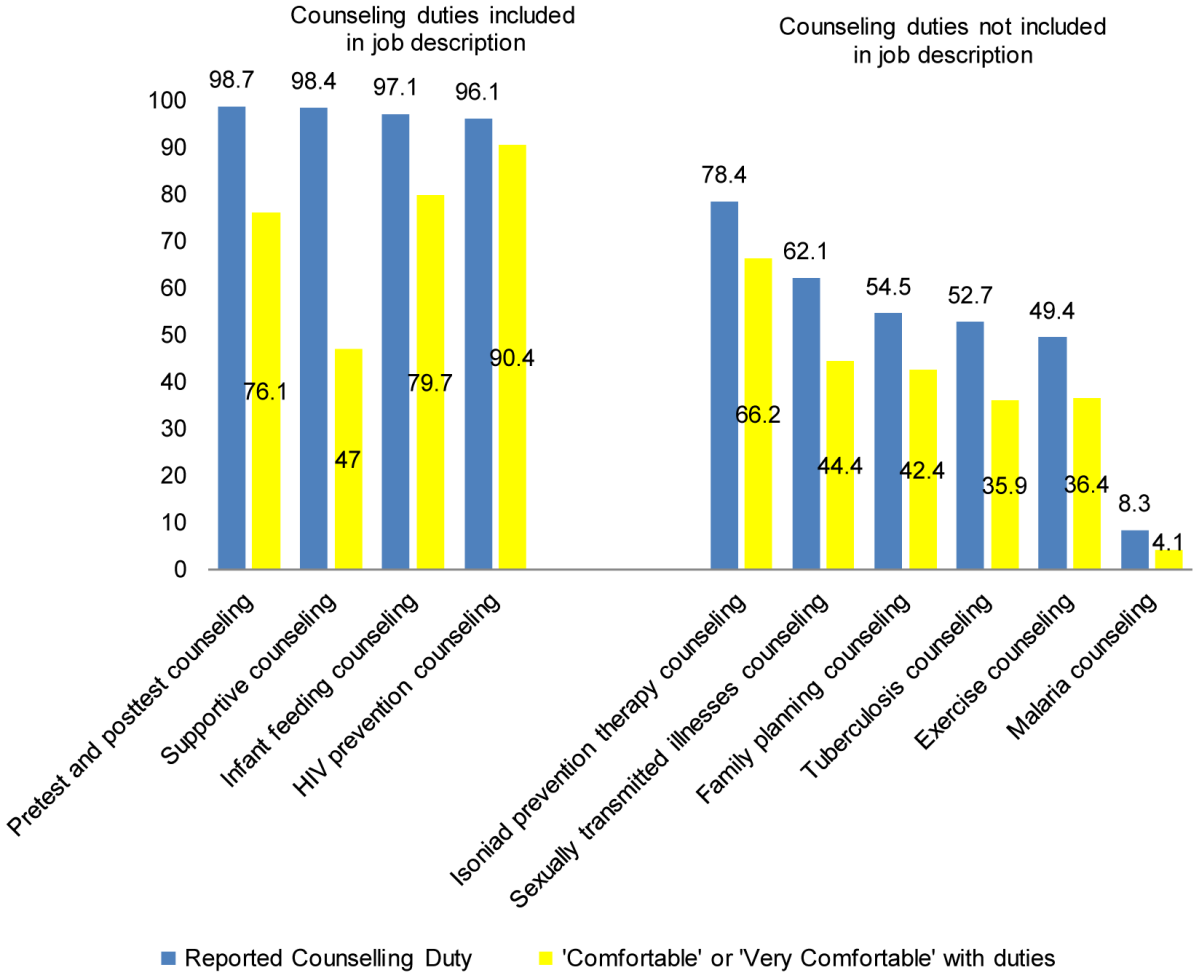
Percent reporting counseling duties and the percent'comfortable/very comfortable' with these duties (n = 385 lay counselors).

Figure 2 provides information on counseling activities reported by the lay counselors in the survey. The lay counselors reported providing counseling related to a number of different areas, including those within and outside of their job description. Counseling duties outside of the job description included counseling on isoniazid prevention therapy, sexually transmitted infections, family planning, tuberculosis, exercise, and malaria. The percent of lay counselors who reported being comfortable in performing counseling duties varied from 90.4% to 4.1% and were noticeably higher for duties that were specifically included in the job description. For duties included in the lay counselors job description, lay counselors felt most comfortable with HIV prevention counseling and least comfortable with supportive counseling, with 90.4% and 47.0% of the lay counselors, respectively, indicating they were comfortable with these duties. In terms of duties not included in the job description, lay counselors felt most comfortable with counseling related to isoniazid prevention therapy and least comfortable with counseling related to malaria, with 68.2% and 4.1% of the lay counselors, respectively, indicating they were comfortable with these duties. Comfort-level ratings on a 5-point scale (1-very uncomfortable, 2 uncomfortable, 3-neither comfortable nor uncomfortable, 4-comfortable, 5- very comfortable) were averaged for counseling duties falling within the job description and for counseling duties outside of the job description. Mean comfort-level ratings were higher (p<0.05) for counseling duties within the job description (3.42±0.84) than those outside of the job description (2.00±1.32). The number of years of experience as a lay counselors was positively correlated (r = 0.14, p<0.06) with comfort rating for counseling duties that are part of the lay counselors' job description; but was not significantly related to comfort rating for counseling duties not included within the job description.

**Figure 3:**
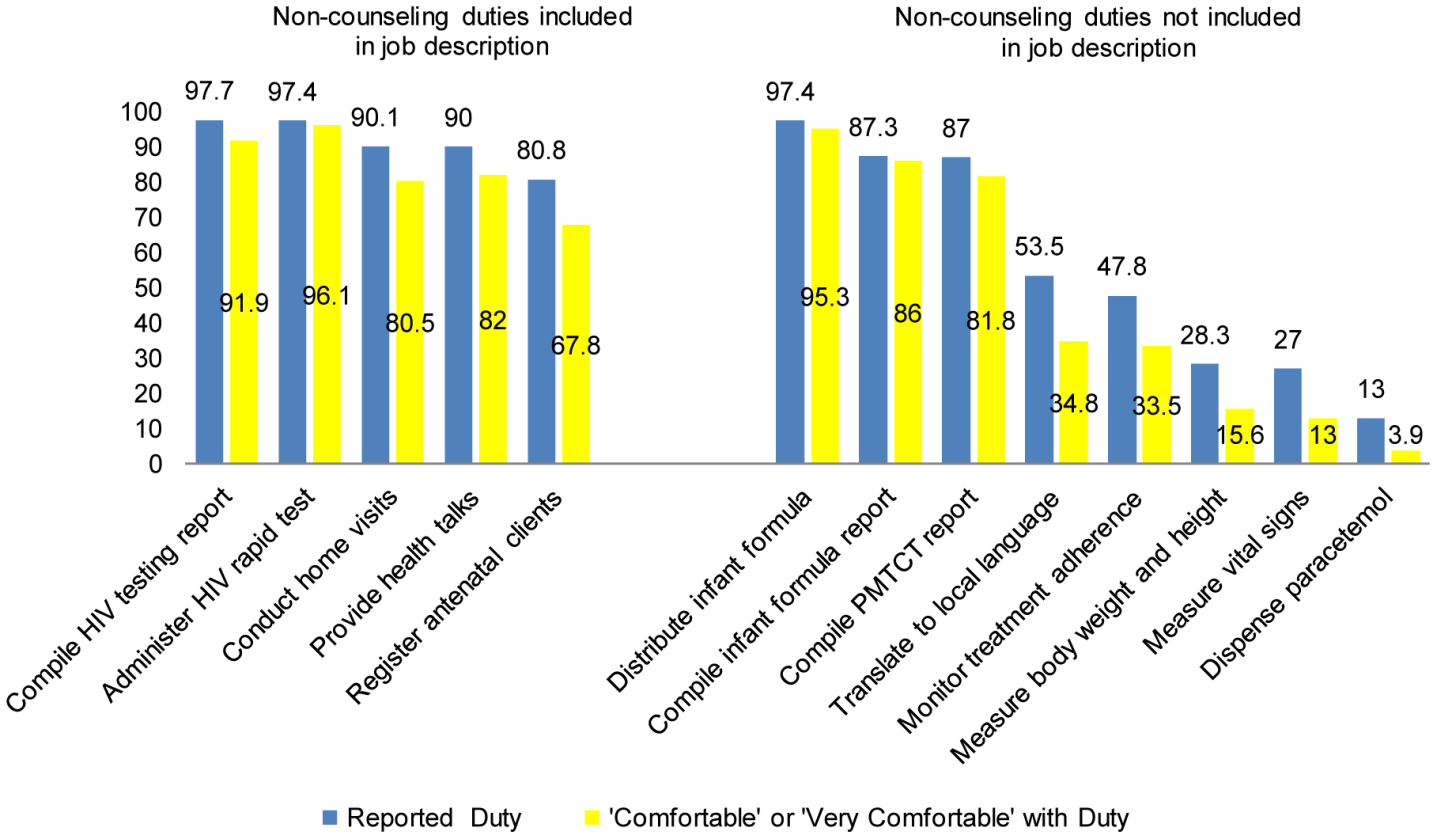
Percent reporting non-counseling duties and the percent ‘comfortable/very comfortable’ with these duties (n = 385 lay counselors).

Figure 3 presents duties other than counseling, which were commonly reported in the survey as being performed by the lay counselors. The percent of lay counselors who reported being comfortable in performing their non-counseling duties within their job description varied from 96.1% for administration of rapid HIV test to 67.8% for registration of antenatal clients. For duties not included in the job description, 82% or more of the lay counselors indicated they were comfortable with distributing infant formula, compiling infant report on infant formula distribution, and compiling PMTCT reports. Mean comfort-level ratings were higher (p<0.001) for non-counseling duties within the job description (4.10±0.86) than those outside of the job description (2.33±0.87). Comfort-level ratings were not significantly correlated with years of experience as a lay counselor for either non-counseling duties included or not included in the job description.

In addition to the duties in Figures 2 and 3, the lay counselors were asked to list any other tasks they carry out. This yielded further activities not in the job description. For example, 10% of the lay counselors reported one or more of the following activities: labeling and transporting blood specimens, clerical duties, and conducting pharmacy related duties including packaging and distribution of TB and ARV drugs. During the FGDs, lay counselors indicated that clinical duties outside of their job description are often a time-consuming part of their workday. As stated by one lay counselor: *‘I might be a lay counselor, but I have really been taught nursing duties by the nurses I work with. I take vital signs, I dispense medication, and I dress wounds and even write reports for the nurses. Sometimes I spend more time doing their duties as opposed to mine”* As another example of activities that go beyond their job description or training, in five of the seven FGDs, lay counselors indicated they were ordering and interpreting CD4 counts and liver function tests (LFTs) and deciding when clients should be referred to the Infectious Disease Control Clinic to initiate ARV treatment. As stated by one lay counselor, *‘ for CD4 results, the nurses are having us do it. Nobody has taught us how to interpret them, but we actually look at them and estimate what they mean. Like if it is 349, we just assume it is the actual CD4 count. sometimes we will find that LFT [liver function test] is elevated and another one is out of range. We don’t know what that means.’*

During FGDs with lay counselors and during interviews with healthcare workers; it was reported that the expansion of the cadre's duties came at the expense of the tasks the lay counselors were hired to perform. Reasons cited for taking on additional duties included: 1) high workload at facilities coupled with insufficient human resources, 2) lack of a widely shared job description defining lay counselors' responsibilities, 3) lack of formal growth opportunities for lay counselors leading them to seek new skills and take on more advanced duties, 4) sharing of tasks with more skilled healthcare workers eventually evolved into a complete shift of duties, 5) additional duties taken on by lay counselors during 'go slow' strikes by health workers were often maintained after the strike, and 6) the expanding national response to HIV.

### Contribution to the healthcare system

As reported in interviews and FGDs, the role of the lay counselors steadily expanded as the national, multi-faceted response to HIV was implemented in Botswana. They contribute to essentially all HIV-related programs, including PMTCT, ARV treatment, HIV testing and counseling, tuberculosis, and isoniazid preventive therapy; as well as several non-HIV related programs such as child welfare clinics. It was reported that lay counselors were essentially conducting all HIV testing for pregnant women, as well as the majority of testing related to routine HIV testing and voluntary counseling and testing at public health facilities. As part of the desk review, reports from the PMTCT program depicting HIV testing trends and program uptake for pregnant women were reviewed, which indicate that both the number of women tested and PMTCT program uptake have steadily increased since the lay counselor cadre was initiated. During national-level interviews, it was reported that lay counselors perform better on HIV proficiency tests and do a better job with documentation for monitoring and evaluation as compared to other cadres of health workers. In addition to testing, lay counselors were the main providers of HIV-related counseling throughout the country at public health facilities. During the interviews with health workers, most of the interviewees (94%) indicated they felt the lay counselors were having a positive impact on the health system. For example, as stated in one national-level interview, *‘I think they should look back with pride at what they have done, for the changes that we have seen with the programs as well as the other responsibilities that have been handed over to them. I accredit them for the fact that they are really making a difference.*’

During interviews, health workers indicated that task shifting of counseling and testing services to the lay counselors was a true accomplishment that had made their workload more manageable. Health workers also indicated that the lay counselors had been instrumental in making HIV a more acceptable issue in communities through outreach activities and by being a part of the communities they serve. During FGDs, lay counselors also reported this as an achievement, indicating that they had a strong rapport with community members that is sometimes hard to achieve by more-skilled health workers.

### Factors that affect the performance of lay counselors

A number of factors were identified that are likely to affect the ability of the lay counselors to perform their work effectively. In the FGDs, lay counselors indicated they felt insufficient resources often hindered the smooth delivery of services to clients (e.g., office space for private counseling sessions, protective clothing, and HIV test kits). Workload was also cited as affecting service delivery; however, there was substantial variation in workload reported by the lay counselors on the survey, with lay counselors reporting from 1 to 50 clients per/day with a mean±SD of 11±7 clients per day (median = 10 clients per day).

Several aspects of the lay counselors' terms of employment were identified as affecting performance, including concerns related to job security, low salary, and insufficient salary progression and career advancement. They reported during the FGDs that the word ‘lay’ in their title adversely affects the way clients and other health workers perceive the services they deliver; this was not, however, observed in the exit interviews. The ability of the lay counselors to perform their job duties was also influenced by the lack of an updated, widely distributed job description delineating the roles and responsibilities of the lay counselors. In their responses to the national survey, only 29% of the lay counselors indicated they had ever received a copy of their job description. As one supervisor stated during the in-depth interviews, ‘*We need their job descriptions to be spelt out clearly, so that it is clear to both them and their supervisors on what they are supposed to be doing.*’

Technical, administrative, and supervisory support, which were assessed as part of the survey, were only cited as satisfactory by 38%, 31%, and 46% of the lay counselors, respectively, which impacts service delivery. While the most recent version of the lay counselors' job description indicated that direct supervision of the cadre was to be provided by a registered nurse/midwife, supervision varied across facilities and districts, with multiple different cadres identified as supervising the lay counselors, including doctors, nurses, PMTCT focal persons, and social workers. Lay counselors commonly reported frustration during the FGDs over the lack of general support from other health workers when they are on leave or off site. As stated by one, *‘when a lay counselor is on leave for a month, testing stops for that period.’* Additionally, the lay counselors reported that they often felt unappreciated within the healthcare system.

A factor positively influencing their ability to perform their duties is the high morale of the lay counselors. Only 14% indicated on the survey they found their work to be unsatisfying. Some of them indicated that the main reason they are still with the cadre is their passion for making a difference in people's lives. Below are several quotes from lay counselors collected in the FGDs:

*‘The people are the reason why we love this job.’*

*‘Babies are born without HIV. This is what keeps us going.’*

*‘In a way I feel like a hero... the community that I am serving really appreciates me.’*

*‘When you look at where you started with a patient and seeing them get better with time...it is so nice to see that.’*

*‘I enjoy mostly the feedback from the clients, appreciating how we assisted them with knowing their status and standing with them until they delivered their HIV-negative child. They tell us that we are doing a good job, and that is fulfilling.’*

Burnout and stress are also serious concerns for lay counselors. As stated by one lay counselor during the FGDs, *‘I can say that we were prepared to do the work but not the consequences of the job.’* During FGDs, lay counselors expressed a need for mechanisms to help them cope with the psychosocial demands related to HIV counseling and testing.

## Discussion

Maintaining a skilled health workforce is a challenge for most countries highly affected by the HIV epidemic. Nevertheless, Botswana has emerged as an international leader in the implementation of innovative approaches to HIV prevention, care, and treatment; establishing the first national ARV and PMTCT programs in Africa.[40], [41] _ENREF_43 Botswana has also been a world leader in the provision of routine HIV testing at public health facilities.[44], [45] The provision of these and other HIV/AIDS programs has led to a very comprehensive package of HIV/AIDS-related prevention, care, and treatment services. This evaluation highlights the role that lay counselors have had in the success of these initiatives as well as the overall health system in the country.

Specifically, the lay counselor cadre has played a key role in the following areas: 1) increasing acceptance of HIV at the community level, 2) serving as the entry point into care and treatment services, and 3) allowing more-skilled health workers to have more time for other tasks. Lay counselors are uniquely positioned to relate to and reach patients as they are integrated into both the community and the health system. This allows them to build trust and acceptability related to HIV services as well as to help mitigate stigma and discrimination. Several other studies have also noted that lay counselors are instrumental in reaching community members.[14], [46] The lay counselors play a key role in implementing national HIV testing and counseling services. Even though the guidelines indicate all clinical staff should be providing HIV testing and counseling services,[47] this study found that lay counselors were the main providers of HIV testing and counseling at public health facilities. Subsequently, the lay counselors have served as the entry point into accessing HIV care, support, and treatment programs throughout the country. Furthermore, the lay counselors have played a critical role in allowing more-skilled health workers, including professional counselors, to focus on duties which require higher levels of training and skill. This has been critical in the successful scale up of a diverse package of HIV services that are delivered with good clinical and program outcomes.

This study provides multiple data sources indicating that the performance of the lay counselors has been very positive, including feedback from health workers and clients. Previously, there has been relatively little examination of the contribution of lay counselors to the health system. One study investigating PMTCT uptake as the program was being initiated in Botswana found that receiving counseling from a lay counselor, instead of a midwife or nurse, was associated with a high client HIV knowledge score.[40] This was likely due to the lay counselor having both dedicated time available and a specific mandate to provide the counseling as well as the high morale of the lay counselors towards their work. Additionally, a recent study in HIV counseling in Botswana that included lay counselors found that counselors expressed a high level of self-perceived effectiveness.[48] The high levels of professional dedication and morale cited in the present study were similar to findings from a recent study in Zambia in which lay counselors were also found to be dedicated and praised by their higher-level cadres.[14]

The success of the lay counselor cadre in Botswana contributes to the growing body of literature highlighting that task shifting can be an effective method to leverage scarce healthcare resources. Importantly, this study also provides insight into how task shifting can be implemented to achieve the greatest impact by providing the opportunity to examine lessons learned from a long-running program. One important lesson learned from this evaluation is the need for task shifting initiatives to grow and mature along with programs. Many of the lay counselors have held their position for five or more years. While a variety of in-service trainings were held for the lay counselors, attention to career progression has been minimal. Career opportunities have been shown to be important for motivation and retention of health workers.[49]

While this study has helped document many of the positive aspects of the lay counselor cadre, it has also highlighted areas of deficiency. A key finding from this evaluation is that the lay counselors were often engaged in tasks that went beyond their formal training and mandate, such as interpreting laboratory results and dispense medications. This can be seen as a positive reflection on the lay counselors' desire to contribute to the health sector and on the confidence instilled by their supervisors. Nevertheless, this leads to questions related to the quality of services they provide as well as concerns regarding legal issues and accountability. Additionally, it limits time available for duties they were hired to carry out. It is essential that steps be taken to ensure that the duties conducted by the lay counselors are aligned with the responsibilities under their purview as well as their training. The need for clearly delineated supervision processes and health worker responsibilities has also been noted in a small study in Cameroon.[50]

In addition to clearly defining roles and responsibilities, this study also highlights the importance of ensuring that quality assurance mechanisms are in place to monitor implementation of the services provided by lay counselors and to improve the quality of these services. Self-reported comfort levels for various tasks did vary, highlighting the need for targeted support. Specifically, the data highlight the need to bolster the lay counselors' ability to comfortably provide supportive counseling and registration for antenatal clients, which were the components of the job description in which the fewest lay counselors reported they were comfortable with conducting. Furthermore, three of the ten items on the multiple-choice test were incorrectly answered by most of the lay counselors. In addition to questions about the appropriate age for administration of HIV antibody test in children and national HIV prevalence, this included one question related to interpretation of discordant HIV-test results. The direct observations of lay counselors also highlighted the need for targeted supportive supervision related to HIV test administration, which is a critical competency for lay counselors, to reinforce test incubation time, interpretation of test results, and data recording. The provision of supportive supervision has been shown to contribute to improvements in the performance of other cadres of health workers and to a more general strengthening of health systems in a number of different countries.[51] The need is highlighted by a recent study in Mozambique in which poor quality of care for duties shifted to non-physician clinicians was identified and led to substantial changes in the scope of work and training for that cadre.[8] Additionally, findings from the WHO-commissioned Study on Task Shifting, found that task shifting was more effective when health workers were provided with lasting and supportive supervision.[2]

Addressing the psychosocial needs of the lay counselors is important to the success of these types of task shifting initiatives. Studies from Tanzania, Kenya, and Zambia have also highlighted that lay counselors often find their work to be rewarding, yet stressful.[14], [46] Testing and counseling can be an emotionally draining job with high levels of psychological stress and burnout. Appropriate management of work-related stressors is important in maintaining motivation and retention of health workers.[49]

This multifaceted study was not without limitations. While the national survey captures information from 94% of lay counselors and the district-level assessment took place in 29% of the health districts; the facility-level interviews, observations, and exit interviews were limited in coverage and may not be generalizable. The lay counselors may have performed better than normal due to the presence of an observer in the observation sessions. For exit interviews, the interviewer was present when the counseling session was taking place, which may have biased client responses. While efforts were made to ensure the lay counselors felt ‘safe’ voicing their opinions during the focus group discussions; social desirability may have biased some responses. Furthermore, the lay counselors in this study were based at public health facilities and conclusions may not be applicable to lay counselors working with community- or faith-based organizations providing community-based testing and counseling services as those individuals would have different training, job duties, and work environments. These results are also not applicable to other cadres identified as responsible for providing testing and counseling services in Botswana, which includes professional counselors, health workers, peer counselors and volunteers. Future work should examine roles and responsibilities across all cadres of health workers to ensure that training is aligned with duties and to identify ways to maximize efficiencies across workflow processes through the redistribution of tasks to improve service delivery.

In conclusion, this evaluation has shown that lay counselors are fulfilling an important role in Botswana's health system. There is, however, a need to address the deficiencies identified. Subsequent to the completion of this study, a process was initiated to address many of the challenges highlighted in this study to ensure an appropriate career trajectory for the cadre as well as to review duties, expand training initiatives, and ensure that these are aligned with the job description. This process involves formally absorbing the cadre in to the government system, which will make the lay counselors permanent and pensionable employees, fully integrate them into the public health sector, and align their roles with that of other paraprofessional cadres. The lay counselors serve as the entry point into HIV care, support, and treatment services. This study highlights the importance of ensuring that lay counselors' roles and responsibilities are clearly delineated and that they are provided with adequate training and support to help deliver comprehensive, compassionate, high quality HIV health services. These data provide guidance on how this, as well as other similar initiatives, can be adjusted to increase performance of task shifting initiatives.

## References

[1] World Health Organization (2006) Task shifting to tackle health worker shortages. Geneva: World Health Organization.

[2] World Health Organization (2008) Task shifting: global recommendations and guidelines: World Health Organization

[3] Swider SM (2002) Outcome effectiveness of community health workers: an integrative literature review. Public Health Nurs 19: 11–20. 11841678 10.1046/j.1525-1446.2002.19003.x

[4] Yong CS (2006) Task substitution: the view of the Australian Medical Association. Med J Aust 185: 27–28. 16813545 10.5694/j.1326-5377.2006.tb00446.x

[5] Horrocks S, Anderson E, Salisbury C (2002) Systematic review of whether nurse practitioners working in primary care can provide equivalent care to doctors. BMJ 324: 819–823. 11934775 10.1136/bmj.324.7341.819 PMC100791

[6] Babigumira JB, Castelnuovo B, Lamorde M, Kambugu A, Stergachis A, et al (2009) Potential impact of task-shifting on costs of antiretroviral therapy and physician supply in Uganda. BMC Health Serv Res 9: 192. 19845963 10.1186/1472-6963-9-192 PMC2770050

[7] Bemelmans M, Van Den Akker T, Ford N, Philips M, Zachariah R, et al (2010) Providing universal access to antiretroviral therapy in Thyolo, Malawi through task shifting and decentralization of HIV/AIDS care. Trop Med Int Health 15: 1413–1420. 20958897 10.1111/j.1365-3156.2010.02649.x

[8] Brentlinger PE, Assan A, Mudender F, Ghee AE, Vallejo Torres J, et al (2010) Task shifting in Mozambique: cross-sectional evaluation of non-physician clinicians' performance in HIV/AIDS care. Hum Resour Health 8: 23. 20939909 10.1186/1478-4491-8-23 PMC2994547

[9] Callaghan M, Ford N, Schneider H (2010) A systematic review of task- shifting for HIV treatment and care in Africa. Hum Resour Health 8: 8. 20356363 10.1186/1478-4491-8-8 PMC2873343

[10] de Wet K, Wouters E, Engelbrecht M (2011) Exploring task-shifting practices in antiretroviral treatment facilities in the Free State Province, South Africa. J Public Health Policy 32 Suppl 1S94–101. 21730997 10.1057/jphp.2011.30

[11] Ivers LC, Jerome JG, Cullen KA, Lambert W, Celletti F, et al (2011) Task-shifting in HIV care: a case study of nurse-centered community-based care in Rural Haiti. PLoS One 6: e19276. 21573152 10.1371/journal.pone.0019276 PMC3089597

[12] McCollum ED, Preidis GA, Kabue MM, Singogo EB, Mwansambo C, et al (2010) Task shifting routine inpatient pediatric HIV testing improves program outcomes in urban Malawi: a retrospective observational study. PLoS One 5: e9626. 20224782 10.1371/journal.pone.0009626 PMC2835755

[13] Morris MB, Chapula BT, Chi BH, Mwango A, Chi HF, et al (2009) Use of task-shifting to rapidly scale-up HIV treatment services: experiences from Lusaka, Zambia. BMC Health Serv Res 9: 5. 19134202 10.1186/1472-6963-9-5 PMC2628658

[14] Sanjana P, Torpey K, Schwarzwalder A, Simumba C, Kasonde P, et al (2009) Task-shifting HIV counselling and testing services in Zambia: the role of lay counsellors. Hum Resour Health 7: 44. 19480710 10.1186/1478-4491-7-44 PMC2692981

[15] Selke HM, Kimaiyo S, Sidle JE, Vedanthan R, Tierney WM, et al (2010) Task-shifting of antiretroviral delivery from health care workers to persons living with HIV/AIDS: clinical outcomes of a community-based program in Kenya. J Acquir Immune Defic Syndr 55: 483–490. 20683336 10.1097/QAI.0b013e3181eb5edb

[16] Shumbusho F, van Griensven J, Lowrance D, Turate I, Weaver MA, et al (2009) Task shifting for scale-up of HIV care: evaluation of nurse-centered antiretroviral treatment at rural health centers in Rwanda. PLoS Med 6: e1000163. 19823569 10.1371/journal.pmed.1000163 PMC2752160

[17] Bland RM, Little KE, Coovadia HM, Coutsoudis A, Rollins NC, et al (2008) Intervention to promote exclusive breast-feeding for the first 6 months of life in a high HIV prevalence area. AIDS 22: 883–891. 18427207 10.1097/QAD.0b013e3282f768de

[18] Rohleder P, Swartz L (2005) 'What I've noticed what they need is the stats': lay HIV counsellors' reports of working in a task-orientated health care system. AIDS Care 17: 397–406. 15832888 10.1080/09540120512331314376

[19] Shetty AK, Mhazo M, Moyo S, von Lieven A, Mateta P, et al (2005) The feasibility of voluntary counselling and HIV testing for pregnant women using community volunteers in Zimbabwe. Int J STD AIDS 16: 755–759. 16303072 10.1258/095646205774763090

[20] Stekelenburg J, Kyanamina SS, Wolffers I (2003) Poor performance of community health workers in Kalabo District, Zambia. Health Policy 65: 109–118. 12849910 10.1016/s0168-8510(02)00207-5

[21] Sherr K, Pfeiffer J, Mussa A, Vio F, Gimbel S, et al (2009) The role of nonphysician clinicians in the rapid expansion of HIV care in Mozambique. J Acquir Immune Defic Syndr 52 Suppl 1S20–23. 19858931 10.1097/QAI.0b013e3181bbc9c0 PMC5568631

[22] Torpey KE, Kabaso ME, Mutale LN, Kamanga MK, Mwango AJ, et al (2008) Adherence support workers: a way to address human resource constraints in antiretroviral treatment programs in the public health setting in Zambia. PLoS One 3: e2204. 18493615 10.1371/journal.pone.0002204 PMC2377331

[23] Bedelu M, Ford N, Hilderbrand K, Reuter H (2007) Implementing antiretroviral therapy in rural communities: the Lusikisiki model of decentralized HIV/AIDS care. J Infect Dis 196 Suppl 3S464–468. 18181695 10.1086/521114

[24] Gabriel AP, Mercado CP (2011) Evaluation of Task Shifting in Community-Based DOTS Program as an Effective Control Strategy for Tuberculosis. ScientificWorldJournal 11: 2178–2186. 22125465 10.1100/2011/984321 PMC3221595

[25] Mafigiri DK, McGrath JW, Whalen CC (2011) Task shifting for tuberculosis control: A qualitative study of community-based directly observed therapy in urban Uganda. Glob Public Health: 1–15. 10.1080/17441692.2011.552067 PMC3603570 21360381

[26] Chibanda D, Mesu P, Kajawu L, Cowan F, Araya R, et al (2011) Problem-solving therapy for depression and common mental disorders in Zimbabwe: piloting a task-shifting primary mental health care intervention in a population with a high prevalence of people living with HIV. BMC Public Health 11: 828. 22029430 10.1186/1471-2458-11-828 PMC3210104

[27] Kengne AP, Fezeu L, Awah PK, Sobngwi E, Mbanya JC (2010) Task shifting in the management of epilepsy in resource-poor settings. Epilepsia 51: 931–932. 20536528 10.1111/j.1528-1167.2009.02414.x

[28] Petersen I, Bhana A, Baillie K (2011) The Feasibility of Adapted Group-Based Interpersonal Therapy (IPT) for the Treatment of Depression by Community Health Workers Within the Context of Task Shifting in South Africa. Community Ment Health J. 10.1007/s10597-011-9429-2 21687982

[29] Petersen I, Lund C, Bhana A, Flisher AJ (2011) A task shifting approach to primary mental health care for adults in South Africa: human resource requirements and costs for rural settings. Health Policy Plan. 10.1093/heapol/czr012 21325270

[30] Chu K, Rosseel P, Gielis P, Ford N (2009) Surgical task shifting in Sub-Saharan Africa. PLoS Med 6: e1000078. 19440533 10.1371/journal.pmed.1000078 PMC2677109

[31] Chu KM, Ford NP, Trelles M (2011) Providing surgical care in Somalia: A model of task shifting. Confl Health 5: 12. 21762491 10.1186/1752-1505-5-12 PMC3152518

[32] De Brouwere V, Dieng T, Diadhiou M, Witter S, Denerville E (2009) Task shifting for emergency obstetric surgery in district hospitals in Senegal. Reprod Health Matters 17: 32–44. 19523580 10.1016/S0968-8080(09)33437-0

[33] Ford N, Chu K, Mills EJ (2011) Safety of task shifting for male medical circumcision in Africa: a systematic review and meta-analysis. AIDS. 10.1097/QAD.0b013e32834f3264 22112602

[34] Dawad S, Jobson G (2011) Community-based rehabilitation programme as a model for task-shifting. Disabil Rehabil 33: 1997–2005. 21291340 10.3109/09638288.2011.553710

[35] Labhardt ND, Balo JR, Ndam M, Grimm JJ, Manga E (2010) Task shifting to non-physician clinicians for integrated management of hypertension and diabetes in rural Cameroon: a programme assessment at two years. BMC Health Serv Res 10: 339. 21144064 10.1186/1472-6963-10-339 PMC3018451

[36] Lekoubou A, Awah P, Fezeu L, Sobngwi E, Kengne AP (2010) Hypertension, diabetes mellitus and task shifting in their management in sub-Saharan Africa. Int J Environ Res Public Health 7: 353–363. 20616978 10.3390/ijerph7020353 PMC2872286

[37] Baiden F, Akanlu G, Hodgson A, Akweongo P, Debpuur C, et al (2007) Using lay counsellors to promote community-based voluntary counselling and HIV testing in rural northern Ghana: a baseline survey on community acceptance and stigma. J Biosoc Sci 39: 721–733. 17207292 10.1017/S0021932006001829

[38] Samb B, Celletti F, Holloway J, Van Damme W, De Cock KM, et al (2007) Rapid expansion of the health workforce in response to the HIV epidemic. N Engl J Med 357: 2510–2514. 18077816 10.1056/NEJMsb071889

[39] Fulton BD, Scheffler RM, Sparkes SP, Auh EY, Vujicic M, et al (2011) Health workforce skill mix and task shifting in low income countries: a review of recent evidence. Hum Resour Health 9: 1. 21223546 10.1186/1478-4491-9-1 PMC3027093

[40] Creek T, Ntumy R, Mazhani L, Moore J, Smith M, et al (2009) Factors associated with low early uptake of a national program to prevent mother to child transmission of HIV (PMTCT): results of a survey of mothers and providers, Botswana, 2003. AIDS Behav 13: 356–364. 17985228 10.1007/s10461-007-9322-8

[41] Wester CW, Bussmann H, Avalos A, Ndwapi N, Gaolathe T, et al (2005) Establishment of a public antiretroviral treatment clinic for adults in urban Botswana: lessons learned. Clin Infect Dis 40: 1041–1044. 15824998 10.1086/428352

[42] UNAIDS (2000) Tools for evaluating HIV voluntary counselling and testing. Geneva.

[43] Ginwalla SK, Grant AD, Day JH, Dlova TW, Macintyre S, et al (2002) Use of UNAIDS tools to evaluate HIV voluntary counselling and testing services for mineworkers in South Africa. AIDS Care 14: 707–726. 12419119 10.1080/0954012021000005533

[44] Steen TW, Seipone K, Gomez Fde L, Anderson MG, Kejelepula M, et al (2007) Two and a half years of routine HIV testing in Botswana. J Acquir Immune Defic Syndr 44: 484–488. 17211281 10.1097/QAI.0b013e318030ffa9

[45] Creek TL, Ntumy R, Seipone K, Smith M, Mogodi M, et al (2007) Successful introduction of routine opt-out HIV testing in antenatal care in Botswana. J Acquir Immune Defic Syndr 45: 102–107. 17460473 10.1097/QAI.0b013e318047df88

[46] Grinstead OA, van der Straten A (2000) Counsellors' perspectives on the experience of providing HIV counselling in Kenya and Tanzania: the Voluntary HIV-1 Counselling and Testing Efficacy Study. AIDS Care 12: 625–642. 11218548 10.1080/095401200750003806

[47] Government of Botswana (2009) National Guidlines: HIV testing and counselling. Ministry of Health.

[48] Tebatso P, Stockton R (2010) A survey of the perceptions of HIV/AIDS counsellors in Botswana. Gaborone, Botswana: Institute of Development Managment.

[49] Willis-Shattuck M, Bidwell P, Thomas S, Wyness L, Blaauw D, et al (2008) Motivation and retention of health workers in developing countries: a systematic review. BMC Health Serv Res 8: 247. 19055827 10.1186/1472-6963-8-247 PMC2612662

[50] Yakam JC, Gruenais ME (2009) Involving new actors to achieve ART scaling-up: difficulties in an HIV/AIDS counselling and testing centre in Cameroon. Int Nurs Rev 56: 50–57. 19239516 10.1111/j.1466-7657.2008.00680.x

[51] Kilminster SM, Jolly BC (2000) Effective supervision in clinical practice settings: a literature review. Med Educ 34: 827–840. 11012933 10.1046/j.1365-2923.2000.00758.x

